# Berberine alleviates visceral hypersensitivity in rats by altering gut microbiome and suppressing spinal microglial activation

**DOI:** 10.1038/s41401-020-00601-4

**Published:** 2021-02-08

**Authors:** Jin-dong Zhang, Jiao Liu, Shi-wei Zhu, Yuan Fang, Ben Wang, Qiong Jia, Hui-feng Hao, John Y. Kao, Qi-hua He, Li-jin Song, Fei Liu, Bao-li Zhu, Chung Owyang, Li-ping Duan

**Affiliations:** 1 Department of Gastroenterology, Peking University Third Hospital, Beijing, 100191 China; 2 Centre of Medical and Health Analysis, Peking University, Beijing, 100191 China; 3 Department of Integration of Chinese and Western Medicine, Key Laboratory of Carcinogenesis and Translational Research (Ministry of Education/Beijing), Peking University Cancer Hospital & Institute, Beijing, 100142 China; 4 Gastroenterology Research Unit, Department of Internal Medicine, University of Michigan, Ann Arbor, MI USA; 5 CAS Key Laboratory of Pathogenic Microbiology and Immunology, Institute of Microbiology, Chinese Academy of Sciences, Beijing, 100101 China

**Keywords:** berberine, microglia, spinal cord, gut microbiome, visceral hypersensitivity, microbiota-gut-brain axis

## Abstract

Accumulating evidence shows that agents targeting gut dysbiosis are effective for improving symptoms of irritable bowel syndrome (IBS). However, the potential mechanisms remain unclear. In this study we investigated the effects of berberine on the microbiota-gut-brain axis in two rat models of visceral hypersensitivity, i.e., specific pathogen-free SD rats subjected to chronic water avoidance stress (WAS) and treated with berberine (200 mg· kg^−1^ ·d^−1^, ig, for 10 days) as well as germ-free (GF) rats subjected to fecal microbiota transplantation (FMT) from a patient with IBS (designated IBS-FMT) and treated with berberine (200 mg· kg^−1^ ·d^−1^, ig, for 2 weeks). Before the rats were sacrificed, visceral sensation and depressive behaviors were evaluated. Then colonic tryptase was measured and microglial activation in the dorsal lumbar spinal cord was assessed. The fecal microbiota was profiled using 16S rRNA sequencing, and short chain fatty acids (SCFAs) were measured. We showed that berberine treatment significantly alleviated chronic WAS-induced visceral hypersensitivity and activation of colonic mast cells and microglia in the dorsal lumbar spinal cord. Transfer of fecal samples from berberine-treated stressed donors to GF rats protected against acute WAS. FMT from a patient with IBS induced visceral hypersensitivity and pro-inflammatory phenotype in microglia, while berberine treatment reversed the microglial activation and altered microbial composition and function and SCFA profiles in stools of IBS-FMT rats. We demonstrated that berberine did not directly influence LPS-induced microglial activation in vitro. In both models, several SCFA-producing genera were enriched by berberine treatment, and positively correlated to the morphological parameters of microglia. In conclusion, activation of microglia in the dorsal lumbar spinal cord was involved in the pathogenesis of IBS caused by dysregulation of the microbiota–gut–brain axis, and the berberine-altered gut microbiome mediated the modulatory effects of the agent on microglial activation and visceral hypersensitivity, providing a potential option for the treatment of IBS.

## Introduction

Functional gastrointestinal disorders (FGIDs) are disorders of gut–brain interactions according to the ROME IV criteria [1]. As the most common FGID in clinical practice, irritable bowel syndrome (IBS) is a chronic heterogeneous disorder originating from multiple factors, including the brain-to-gut and gut-to-brain pathways [2, 3]. Emerging evidence indicates that IBS is characterized by stress pathway activation [4], low-grade intestinal mucosal inflammation, intestinal barrier impairment [5, 6], visceral hypersensitivity [7], altered gastrointestinal motility [8], and gut dysbiosis [9–11], and the concept of microbiota–gut–brain axis dysregulation has been proposed to explain the pathological mechanisms of IBS in recent years [12].

The immune system is a critical component of the microbiota-gut-brain axis and thus a vital pathway through which the gut microbiota influences the central nervous system (CNS) [13]. Accordingly, systematic and local immune markers have been found to be associated with IBS symptoms [14, 15]. Microglia are resident immune cells in the CNS and can be activated by pathological events, leading to neuroinflammation [16], which is involved in the onset of visceral hypersensitivity [17–19]. Recent studies have found that activation of microglia is involved in certain diseases caused by dysregulation of the microbiota–gut–brain axis [20, 21], and therapeutic methods targeting the gut microbiota have been shown to have protective effects on microglial activation in neuropsychiatric diseases [22–24]. However, it is still unclear whether there is a relationship between the microbiota and microglia in the pathogenesis and treatment of visceral hypersensitivity.

Berberine, the major pharmacological component of the Chinese herb *Coptis chinensis* (Huang-Lian), has been used to treat bacterial diarrhea. Recently, berberine was also shown to be effective in the treatment of colorectal adenoma recurrence, metabolic disorders, and neuropsychiatric disorders [25–27]. Owing to its poor oral bioavailability, berberine has been shown to function via modulation of the gut microbiota [28, 29]. Thus, berberine may have applications in the treatment of microbiota–gut–brain axis disorders, such as IBS, particularly for patients with concurrent IBS and psychiatric disorders.

In this study, we aimed to evaluate the role of berberine in modulating microglial activation and the gut microbiome in two models of visceral hypersensitivity, i.e., one induced by chronic water avoidance stress (WAS) and the other induced by fecal microbiota transplantation (FMT) from a patient with IBS (designated IBS-FMT) into germ-free (GF) rats, and to explore the potential therapeutic value of berberine for the treatment of IBS.

## Materials and methods

### Animals and experimental design

Male specific pathogen-free (SPF) and GF Sprague–Dawley rats (6 weeks old) were bred at the Department of Laboratory Animal Science, Peking University Health Science Center in Beijing, China. All animals were maintained on a 12-h light/dark cycle at a constant temperature (23 ± 2 °C) and humidity (63% ± 2%), and autoclaved food and water were provided ad libitum. SPF rats were housed two per cage in individually ventilated cages, and GF rats were kept individually in flexible film isolators. The study was divided into two parts. In the first experiment, two groups of SPF rats were exposed to WAS and received vehicle or berberine. The control group underwent sham WAS and received vehicle gavage. To verify that the preventive effects on visceral hypersensitivity were dependent on the berberine-induced alteration of the microbiota, we collected stools from vehicle- or berberine-treated stressed rats, transferred them to GF rats, and challenged the recipient rats with acute WAS after colonization for 5 weeks (Supplementary Fig. 1a). In the second experiment, after 7 days of acclimatization, GF rats were divided into three groups; the first group received FMT from a healthy donor, whereas groups 2 and 3 received FMT from a patient with diarrhea-predominant IBS (IBS-D). After colonization for 14 days, groups 1 and 2 received vehicle gavage, whereas group 3 received berberine gavage for 14 days (Supplementary Fig. 1b). All protocols were approved by the Laboratory Animal Welfare Ethics branch of the Biomedical Ethics Committee of Peking University (approval no. LA2016230).

### WAS procedure

Repeated WAS procedures were conducted as described previously [30]. Rats were placed on a block (10 cm × 8 cm × 8 cm) affixed to the center of the floor of a Plexiglas tank (45 cm × 25 cm × 25 cm) filled with 25 °C fresh water to a level 1 cm below the top of the block. The rats were kept on the block for 1 h each day (5:00–6:00 p.m.) for 10 consecutive days. The rats in the sham group were placed on an identical platform in a container without water for 1 h each day for 10 days. The fecal pellets in the container were counted at the end of each 1-h WAS or sham session.

### Collection of human stool samples

IBS-D was diagnosed according to ROME III criteria. The exclusion criteria included clinically significant major systemic disease; major mental illness; pregnancy or lactation in women; history of cancer and inflammatory gastrointestinal diseases; and use of probiotics and antibiotics within 1 month prior to sample collection. Stool samples were collected from each patient, immediately transported to the laboratory in a container with an ice pack, and stored at −80 °C until use. All individuals who participated in this study provided written informed consent, and the protocol was approved by the Peking University Third Hospital Medical Science Research Ethics Committee (approval no. 2013-112).

### FMT procedures

To perform the first FMT experiment, samples from WAS rats and stressed rats that received berberine treatment were collected and mixed by combining an equal weight of each sample. One gram of mixed stool from each group was suspended in 50 mL sterile phosphate-buffered saline (PBS) containing 20% (v/v) glycerol. An aliquot of 2 mL of the suspension was orally administered to GF rats, and the rest of the suspension was splashed onto the bedding.

To perform the second FMT experiment, we used stool samples from one treatment-naïve patient with IBS-D and one healthy control; the samples were sequenced in a previous clinical study [31] (Supplementary Table 1). Briefly, 600 mg frozen stool from each human donor was suspended in 30 mL sterile PBS containing 20% (v/v) glycerol. The GF rats were randomized into three groups and colonized by oral gavage with 2 mL suspension and inoculation of the bedding.

### Evaluation of visceral sensitivity

Graded colorectal distension (CRD) tests were performed using a well-established and validated method [32]. Briefly, under light isoflurane anesthesia, a flexible balloon made of a polyethylene plastic bag (4–5 cm) was inserted into the distal colon, with its end 1 cm proximal to the anus. After the balloon was inserted into the distal colon, the rats were placed in a Plexiglas cage for 20 min before the CRD test was initiated. Five series of CRD tests were performed in different orders as follows: 20, 40, 60, and 80 mmHg (series 1); 40, 60, 80, and 20 mmHg (series 2); 60, 80, 20, and 40 mmHg (series 3); 80, 20, 40, and 60 mmHg (series 4); and 20, 40, 60, and 80 mmHg (series 5). Each distension lasted for 20 s, with a 4-min interstimulus interval. During each distention, abdominal withdrawal score (AWR) was scored using the following scale: score 1, the rat became immobile during the CRD and occasionally clinched its head at the onset of the stimulus; score 2, a mild contraction in the abdominal muscles was observed, but the rat did not lift its abdomen off the platform; score 3, a strong contraction of the abdominal muscles was observed, and the rat lifted its abdomen off the platform; and score 4, severe contraction of the abdominal muscles was manifested by body arching, and the rat lifted its pelvic structures off the platform [33].

### Depressive behavior tests

Rat behaviors were tested using the sucrose preference test (SPT) [34] and forced swimming test (FST) [35]. For the modified SPT, the rats were individually housed and habituated to two leak-resistant water bottles, one containing pure water and the other containing a 1% sucrose solution, for 48 h. To exclude the interference of position preference to the test results, we exchanged the locations of the two bottles after the first 24 h. After 48 h of habituation, both bottles were removed 6 h prior to testing, and each rat was then given ad libitum access to pure and sucrose water for 1 h, with the bottle location being exchanged after 30 min. The bottles were weighed before and after testing, and sucrose preference was expressed as a fraction (%) of sucrose water consumption divided by total consumption of pure and sucrose water. For the FST, each rat was placed in a Plexiglas cylinder (diameter, 20 cm; height, 50 cm) containing 30 cm double distilled water (25 ± 2 °C) and was videotaped for 5 min. After the swim session, the rat was dried with a towel and returned to its home cage. The water was replaced between each animal. Immobility was defined as the absence of motion except for movements required to maintain the rat’s head above water.

### Serum corticotropin-releasing hormone (CRH) measurement

Upon euthanasia, blood was withdrawn from the apex cordis and placed in a sterile microtube. After centrifugation at 4 °C and 1850  × *g* for 15 min, the supernatant was extracted and stored at −80 °C until use. A radioimmunoarray kit (cat. no. HY-10175; Sinoukbio, Beijing, China) was used to determine CRH concentrations according to the manufacturer’s instructions.

### Immunohistochemical staining

Distal colon tissues were embedded in paraffin and cut into 5-μm-thick sections. After dewaxing and rehydration, the sections were soaked in sodium citrate buffer and heated for antigen retrieval and then incubated with goat serum to block nonspecific binding sites. The slides were then incubated with an anti-tryptase antibody (dilution: 1:500; cat. no. ab2378; Abcam, Cambridge, UK) or anti-mucin2 (Muc2) antibody (dilution: 1:100; cat. no. sc-7314; Santa Cruz Biotechnology, Santa Cruz, CA, USA) at 4 °C overnight, followed by incubation with horseradish peroxidase-conjugated secondary antibodies for 30 min. The sections were developed using a diaminobenzidine substrate kit (cat. no. ZLI-9034; zsBio, Beijing, China). Images were obtained with a Nano Zoomer scanning device (Hamamatsu, Shizuoka, Japan). Tryptase-positive cells were counted, and the intensity of Muc2 expression in the colonic tissues was determined in 3–5 random fields from each section and at least three sections from each rat using Image-Pro Plus software.

### Immunofluorescence staining and three-dimensional reconstruction of microglia

Animals were sedated and transcardially perfused with normal saline solution. The brains and spinal cords were removed and fixed in 4% (w/v) paraformaldehyde. Three 35-μm parasagittal sections of the brain and three cross-sectional sections of the L6-S1 spinal cord were incubated with an anti-Iba-1 antibody (dilution: 1:200; cat. no. 019−19741; Wako, Osaka, Japan) at 4 °C overnight, followed by incubation with an Alexa Fluor 488-conjugated secondary anti-rabbit antibody (dilution: 1:500; cat. no. A-11008; Invitrogen, Carlsbad, CA, USA). The nuclei were counterstained with Hoechst 33342 (cat. no. 94403; Sigma, St. Louis, MO, USA). Section imaging was performed with a Leica SP8 confocal laser scanning microscope with a 63× objective (Leica Biosystems, IL, USA). The amygdala, prefrontal cortex, hippocampus, and L6–S1 dorsal spinal cord were selected based on previous studies showing the relationships between these regions and visceral hypersensitivity [36, 37]. The number of Iba-1-positive cells was assessed by immunofluorescence staining and quantified using ImageJ. The data are presented as the mean number of cells per 0.01 mm^2^.

For three-dimensional reconstruction, Z stacks were imaged with 1-μm steps in the z direction with a resolution of 1024  × 1024 pixels and analyzed using IMARIS software (Bitplane). Semiautomated reconstruction of microglial cell bodies and processes was performed, and the area, volume, total process length, number of branch points, number of segments, and number of terminal points were quantified. In addition, automated Sholl analysis was also performed on each cell using concentric spheres with radii that increased by 1 μm per step, and the number of intersections at each concentric sphere was analyzed. Two to three cells per slice per region from six animals per group were analyzed.

### Fecal microbial DNA extraction

Fecal pellets were collected and frozen at −80 °C until use. Microbial DNA was extracted using an OMEGA-soil DNA Kit (Omega Bio-Tek, GA, USA). The V1–V3 regions of the 16S rRNA gene were amplified by PCR (95 °C for 3 min; 25 cycles at 95 °C for 30 s, 55 °C for 30 s, and 72 °C for 45 s; and a final extension at 72 °C for 10 min) with primers (338 forward primer: 5′-ACTCCTACGGGAGGCAGCAG-3′; 806 reverse primer: 5′-GGACTACHVGGGTWTCTAAT-3′) [38].

### Illumina MiSeq sequencing

Amplicons were extracted from 2% agarose gels and purified using an AxyPrep DNA Gel Extraction Kit (Axygen Biosciences, Union City, CA, USA) according to the manufacturer’s instructions and quantified using QuantiFluor-ST (Promega, USA). Purified amplicons were pooled in equimolar amounts and paired-end sequenced on an Illumina MiSeq platform (Illumina, San Diego, CA, USA) according to standard protocols. Sequencing libraries were constructed using a TruSeq DNA Sample Preparation Kit (Illumina).

### 16S rRNA sequence analysis

Operational taxonomic units (OTUs) were clustered with a 97% similarity cutoff using UPARSE (version 7.1 http://drive5.com/uparse/), and chimeric sequences were identified and removed using UCHIME. The taxonomy of each 16S rRNA gene sequence was analyzed by RDP Classifier (http://rdp.cme.msu.edu/) against the SILVA (SSU115) 16S rRNA database using a confidence threshold of 70% [39]. The within-sample (α) diversity was calculated using Chao1 and Shannon indexes based on the OTU profiles to estimate the richness and diversity of the samples. Weighted principal coordinate analysis was performed based on UniFrac distances to measure community clustering. Predictions regarding the functions of the microbiome were based on 16S rRNA-derived OTUs using PICRUSt.

### Targeted metabolomics

Approximately 100 mg stool was homogenized in 1 mL of 50% aqueous acetonitrile and centrifuged. For derivatization, 40 μL supernatant was mixed with 20 μL of 200 mM 3-nitrophenylhydrazine and 20 μL of 120 mM N-(3-dimethylaminopropyl)-N′-ethylcarbodiimide·HCl-6% pyridine solution. The mixture was reacted at 40 °C for 30 min and then diluted to 800 μL with 10% aqueous acetonitrile. Ten microliters of the solution was injected for measurement. An Ultimate 3000 RSLC system (Dionex Inc., Amsterdam, The Netherlands) coupled to a TSQ Quantiva Ultra triple-quadrupole mass spectrometer (Thermo Fisher, Waltham, MA, USA) was used for short-chain fatty acid (SCFA) detection, and the final data were processed using Xcalibur 3.0.63 software (Thermo Fisher). The data are expressed as mg SCFA/g feces.

### In vitro experiment

Murine BV2 cells were maintained in Dulbecco’s modified Eagle’s medium (DMEM)/F-12 containing 10% fetal bovine serum. At the time of harvest, the cells were washed with PBS, trypsinized, centrifuged, and resuspended in serum-free DMEM; then, the cells were seeded in plates. The cells were treated with various concentrations of berberine (0, 0.1, 0.3, 1, 3, 10, or 30 μM) for 30 min and then stimulated with lipopolysaccharide (LPS; 100 ng/mL) for 24 h. Microscopy was used to observe the morphology of the cells [40].

### Statistics

Abdominal withdrawal reflex scores were analyzed using two-way analysis of variance (ANOVA) with CRD pressure and treatment as factors followed by least significant difference (LSD) posthoc tests. The data for fecal pellet output (FPO) number and SCFA content were analyzed using one-way ANOVA followed by LSD posthoc tests or unpaired Student’s *t* tests if only two groups were compared. Bacterial relative abundances were analyzed by Wilcoxon tests, and the relationships between microglial morphological parameters and alterations in microbial OTUs were analyzed by Pearson’s correlation tests. The results are expressed as the means ± standard errors of the means. Differences between groups were considered significant when the *P* values were < 0.05. Statistical analyses were performed with SPSS version 20.0 (SPSS, Chicago, IL, USA).

## Results

### Berberine prevented stress-induced visceral hypersensitivity and microglial activation

**Fig. 1:**
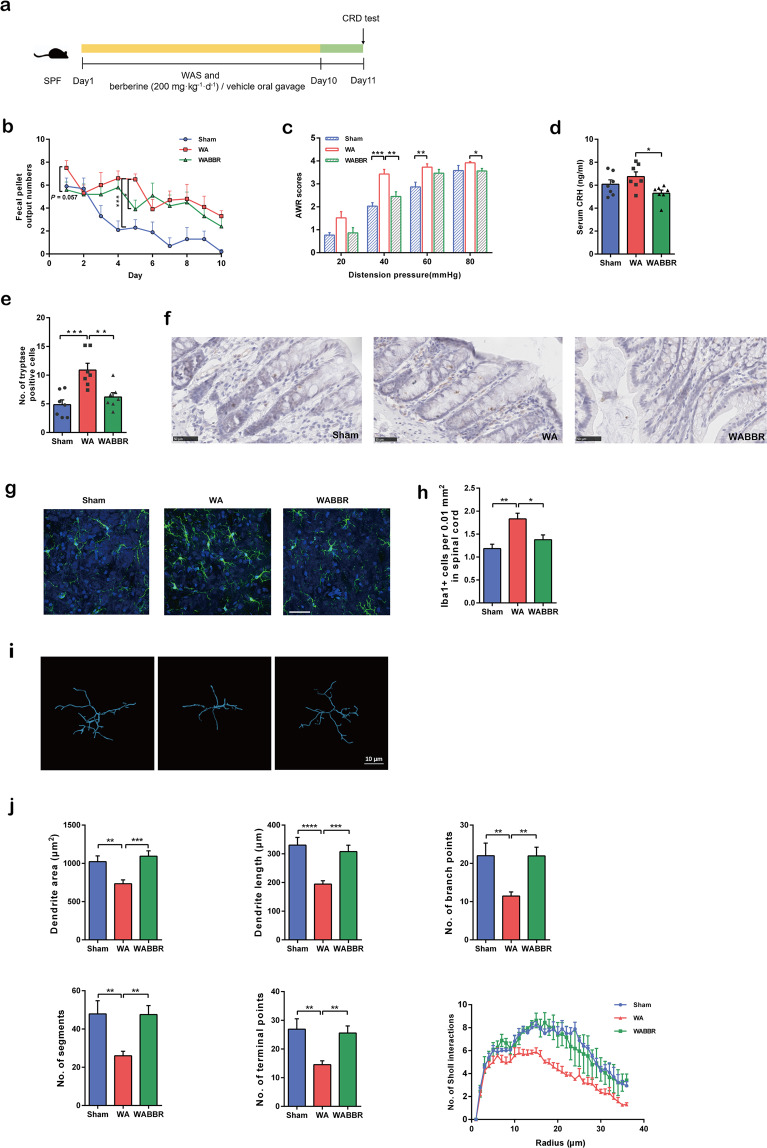
Berberine prevented stress-induced visceral hypersensitivity and microglial activation. **a** Design of the WAS and berberine treatment experiments. *n* = 10/group. **b** Fecal pellet output number in the container after each 1-h WAS. *n* = 10/group. **c** Abdominal withdrawal reflex scores in response to CRD. *n* = 10/group. **d** Levels of serum CRH. *n* = 7/group. **e** Number of tryptase-positive cells in the distal colonic mucosal tissues. **f** Immunohistochemistry showing tryptase-positive cells in the distal colon. *n* = 7/group. **g** Representative images of Iba-1-stained microglia in the dorsal lumbar spinal cord. **h** Number of Iba-1-positive cells in the dorsal lumbar spinal cord. **i** Representative three-dimensional reconstructions of Iba-1-stained microglia residing in the dorsal lumbar spinal cord. **j** Morphological parameters of microglia. *n* = 3/group. *P < 0.05, **P < 0.01, ***P < 0.001, ****P < 0.0001.

To investigate the effects of berberine on mitigating visceral hypersensitivity and activation of microglia, we first utilized the WAS model, a validated method to induce visceral hypersensitivity (Fig. 1a). During the experiment, FPO number was significantly higher in stressed rats than in the sham group, and oral gavage of berberine prevented the increase in FPO number (particularly on days 1 and 5; Fig. 1b). In the CRD tests, berberine significantly attenuated the increased AWR scores of the stressed rats at 40 and 80 mmHg (Fig. 1c). Although we found no significant differences in serum CRH levels between the sham and stressed groups, berberine significantly decreased the levels of this stress-associated hormone (Fig. 1d). Mast cell activation is a ubiquitous mechanism observed in patients with IBS, and we therefore detected the expression of tryptase in distal colonic mucosal tissues. Our results showed that the number of tryptase-positive cells was significantly lower in the berberine-treated group than in the vehicle-treated group (Fig. 1e, f).

In the WAS model, stressed rats showed increased numbers of Iba-1-positive cells in the amygdala and lumbar spinal cord (Fig. 1g, h, Supplementary Table 2) and exhibited Iba-1-positive cells with an ameboid phenotype and decreased morphological parameters; these changes were inhibited by berberine administration (Fig. 1i, j, Supplementary Table 3).

### Berberine altered the gut microbiota in stressed rats

**Fig. 2:**
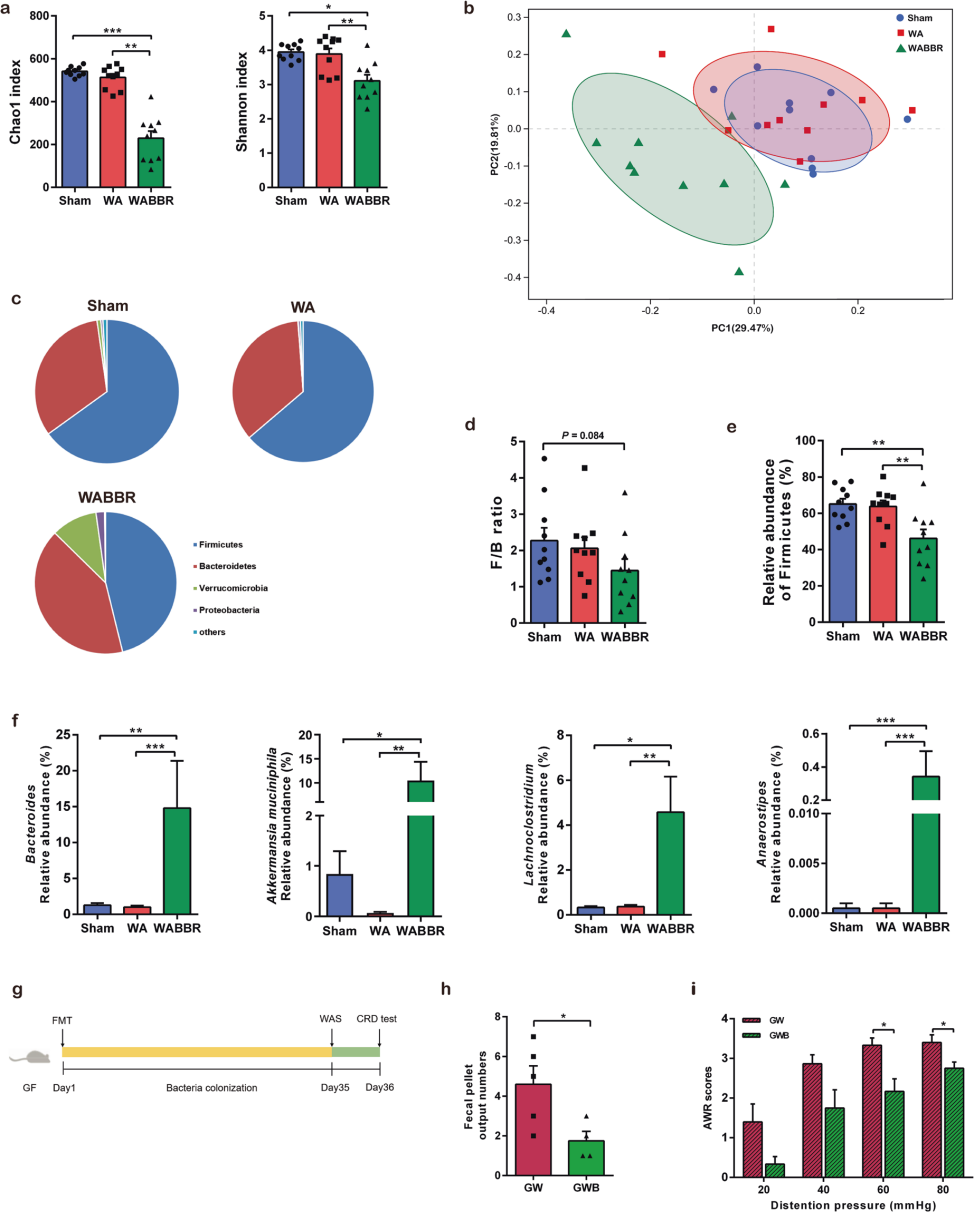
Berberine altered the gut microbiota in stressed rats. **a** Chao1 and Shannon indexes indicative of community richness and diversity. *n* = 10/group. **b** Principal coordinate analysis based on weighted UniFrac distances of the relative abundances of bacterial OTUs after berberine treatment. *n* = 10/group. **c** Taxonomic distributions of bacteria at the phylum level. *n* = 10/group. **d** The F/B ratio in each group. *n* = 10/group. **e** Relative abundance of Firmicutes in each group. *n* = 10/group. **f** Relative abundances of selected genera enriched by berberine. *n* = 10/group. **g** Design of the FMT experiment. *n* = 5 (GW); *n* = 4 (GWB). **h** Fecal pellet output number in the container after acute WAS. **i** Abdominal withdrawal reflex scores in response to CRD. *P < 0.05, **P < 0.01, ***P < 0.001.

To characterize the effects of berberine on the gut microbiota in the stressed model, we collected fecal samples at the end of the experiment (day 11, immediately before the CRD test) and performed 16S rRNA gene sequencing. Decreased α-diversity was observed in the berberine-treated group (Fig. 2a), suggesting that berberine induced the loss of certain bacterial taxa. Principal coordinate analysis revealed that berberine dramatically altered the gut microbial profile (Fig. 2b). At the phylum level, berberine increased the relative abundance of Verrucomicrobia (Fig. 2c) and reduced the Firmicutes/Bacteroidetes (F/B) ratio (Fig. 2d), predominantly by decreasing the relative abundance of Firmicutes (Fig. 2e). Enriched genera following berberine treatment included *Bacteroides*, *Lachnoclostridium*, *Akkermansia*, and *Anaerostipes* (Fig. 2f). Consistent with the increased abundance of *Akkermansia*, which is a key bacterium for the degradation and production of mucin in the mucus layer, we found that Muc2 protein expression in colonic tissues was higher in berberine-treated rats than in the other two groups (Supplementary Fig. 2). These results revealed that the composition of the gut microbiota was substantially altered in response to berberine treatment.

### Alleviation of visceral hypersensitivity was associated with berberine-dependent alterations in the microbiota in stressed rats

To investigate whether the berberine-altered microbiota contributed to the observed reductions in intestinal hypermotility and visceral hypersensitivity in the WAS model, GF rats were colonized with fecal samples from stressed SPF rats that received either berberine or vehicle treatment (Fig. 2g). Fecal samples from donor rats from each group were pooled and transferred to GF rats. After colonization for 5 weeks, the recipient rats were challenged with acute WAS. The FPO number was significantly lower in GF rats that received the berberine-altered microbiota (Fig. 2h). Moreover, compared with those that received the microbiota of vehicle-treated stressed rats, GF rats colonized with the berberine-altered microbiota showed reduced AWR scores in the CRD test (Fig. 2i). These data demonstrated that the alterations in the gut microbiota induced by berberine mediated its protective effects on intestinal hypermotility and visceral hypersensitivity in the WAS model.

### Berberine prevented IBS-FMT-induced visceral hypersensitivity in recipient GF rats

**Fig. 3:**
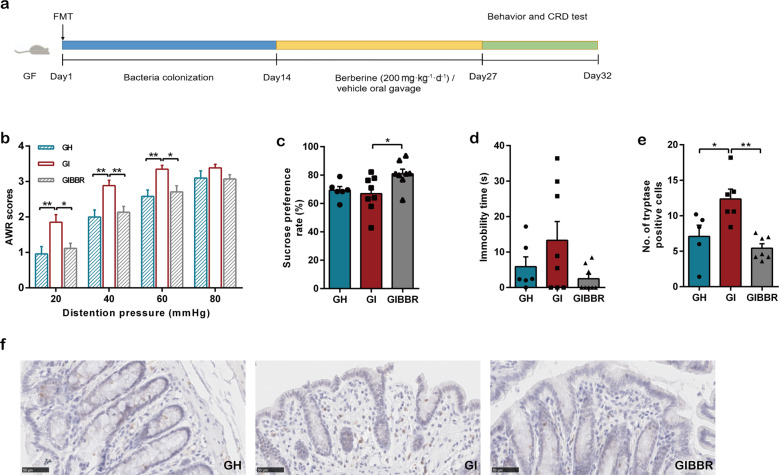
Transplantation of stool derived from an IBS patient induced visceral hypersensitivity, which was prevented by berberine. **a** Design of the FMT experiment. *n* = 12 (GH); *n* = 14 (GI, GIBBR). **b** Abdominal withdrawal reflex scores in response to CRD. *n* = 12 (GH); *n* = 14 (GI, GIBBR). **c** Sucrose preference rates and **d** immobility time after bacterial colonization and berberine treatment. *n* = 6 (GH); *n* = 8 (GI, GIBBR). **e** Number of tryptase-positive cells in distal colonic mucosal tissues. **f** Immunohistochemistry showing tryptase-positive cells in the distal colon. *n* = 5 (GH); *n* = 6 (GI); *n* = 7 (GIBBR). *P < 0.05, **P < 0.01.

IBS is a heterogeneous disorder with different pathological factors, including the brain–gut and gut–brain pathways. Previous studies have revealed that a subset of patients with IBS show differences in microbial structures compared with healthy controls, whereas other patients with IBS have normal signatures [9, 31, 41], and that the altered fecal microbiota from patients with IBS can induce intestinal and behavioral manifestations in GF animals [42, 43]. To further verify the inhibitory effects of berberine on microglial activation through modulation of the microbiota–gut–brain axis, we collected stool samples from one patient diagnosed with new-onset, treatment-naïve IBS-D and one healthy individual as a control. We then transplanted the fecal microbiota into individual groups of GF rats via oral gavage and inoculation of the bedding (IBS-FMT and HC-FMT, respectively). After colonization for 2 weeks, the recipient rats received berberine or vehicle administration for another 2 weeks (Fig. 3a). In the CRD test, we observed that the AWR scores of the IBS-FMT GF rats (GI) were significantly increased compared with those of the HC-FMT GF rats (GH) and that this change in scores was reversed by berberine treatment (GIBBR; Fig. 3b). Although there were no significant differences in sucrose preference rate and immobility time in the behavior tests between rats that received the HC microbiota and those that received the IBS microbiota, berberine increased the rate of sucrose consumption in IBS-FMT rats and tended to decrease immobility time (Fig. 3c, d), suggesting that berberine alleviated depressive behaviors. Furthermore, the number of tryptase-positive cells in the colonic tissues of IBS-FMT rats was higher than that in the colonic tissues of HC-FMT rats and was reduced by berberine treatment (Fig. 3e, f).

### IBS-FMT induced microglial activation, which was inhibited by berberine

**Fig. 4:**
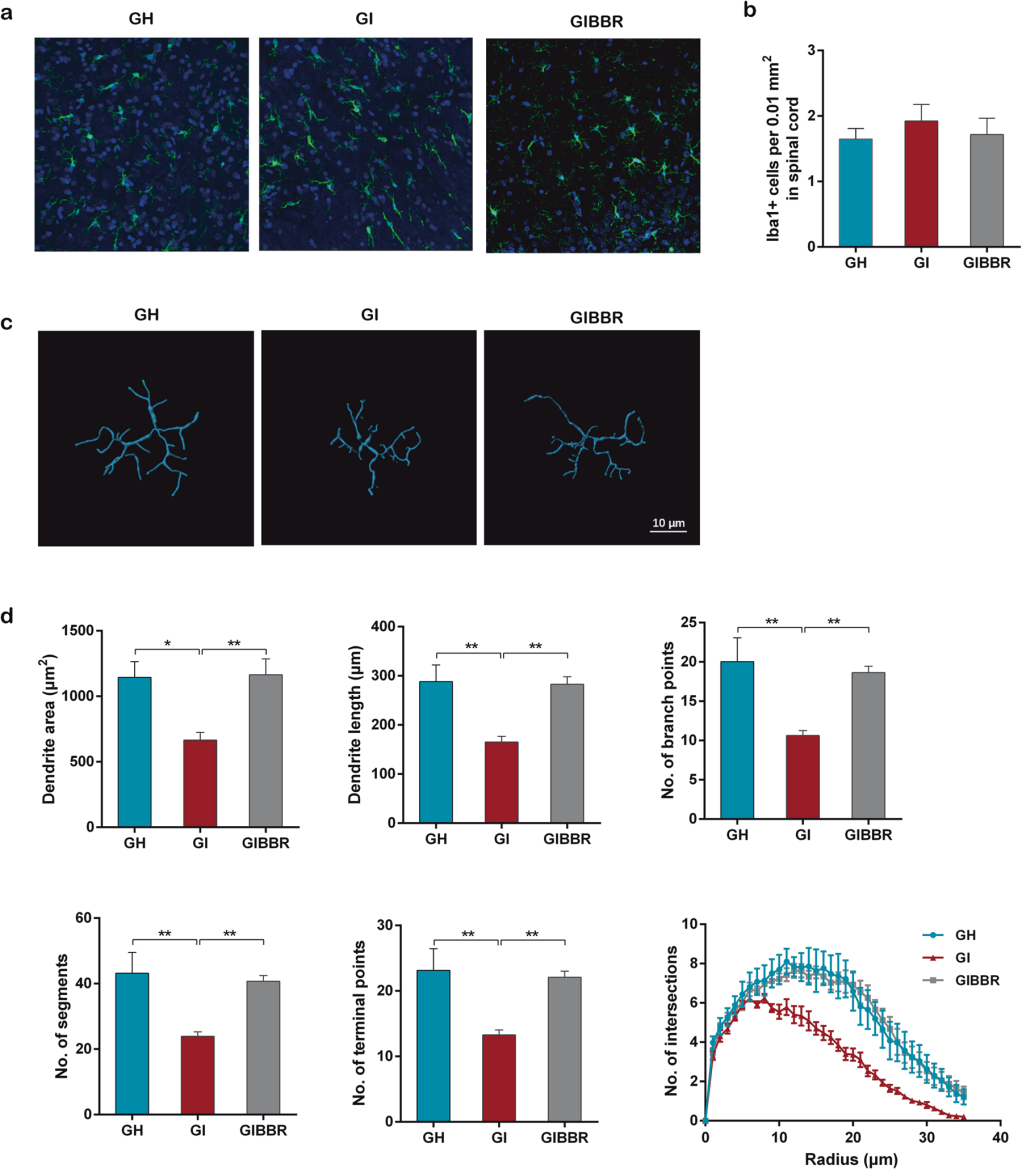
The IBS-derived gut microbiota induced microglial activation, which was inhibited by berberine. **a** Representative images of Iba-1-stained microglia in the dorsal lumbar spinal cord. **b** Number of Iba-1-positive cells in the dorsal lumbar spinal cord. **c** Representative three-dimensional reconstructions of Iba-1-stained microglia residing in the dorsal lumbar spinal cord. **d** Morphological parameters of microglia. *n* = 6/group. *P < 0.05, **P < 0.01.

To investigate whether visceral hypersensitivity induced by IBS-derived gut microbiota was associated with microglial activation and to determine the effects of berberine on microglia, we evaluated the number of activated microglial cells and the morphological phenotype of the microglia. Although there were no significant differences in the numbers of Iba-1-positive cells in the lumbar spinal cord and other selected CNS regions among the three groups (Fig. 4a, b, Supplementary Table 4), microglia from IBS-FMT rats showed significant changes in cell morphology, including decreases in dendritic area, dendritic length, the number of branch points, the number of segments, the number of terminal points, and interactions. Berberine treatment prevented these morphological changes (Fig. 4c, d, Supplementary Table 5).

### Berberine altered the structure and composition of the IBS-derived gut microbiota

**Fig. 5:**
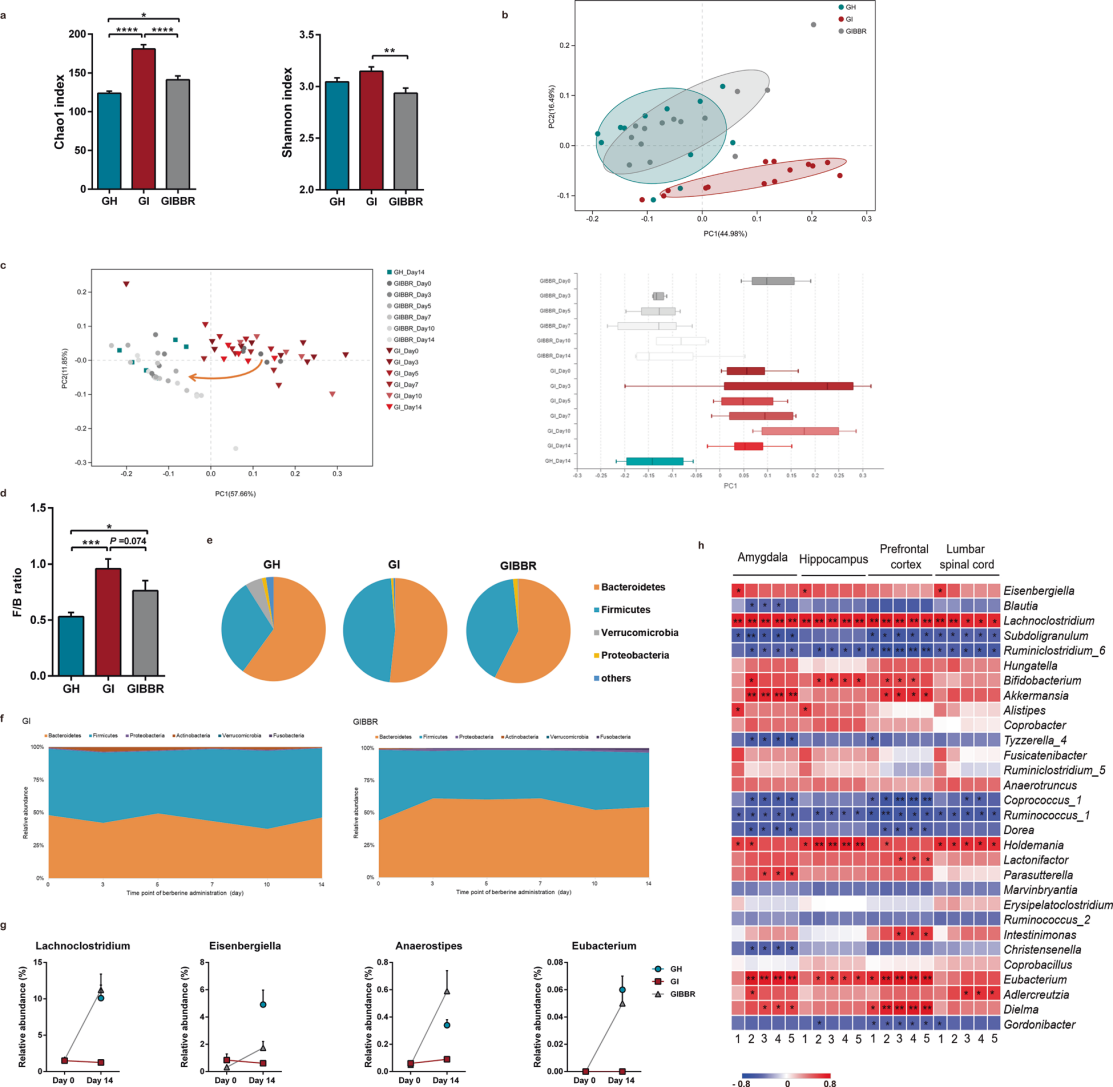
Berberine altered the structure and composition of the IBS-derived gut microbiota. **a** Chao1 and Shannon indexes indicative of community richness and diversity. *n* = 12 (GH); *n* = 14 (GI, GIBBR). **b** Principal coordinate analysis based on weighted UniFrac distances of the relative abundances of bacterial OTUs after berberine treatment. *n* = 12 (GH); *n* = 14 (GI, GIBBR). **c** Weighted UniFrac principal coordinate analysis showing dynamic shifts after berberine or vehicle treatment. *i* = 5–7/group. **d** Comparison of F/B ratios. **e** Bacteria composition at the phylum level. *n* = 12 (GH); *n* = 14 (GI, GIBBR). **f** Dynamic alterations in different phyla in fecal samples during berberine or vehicle treatment. *n* = 5–7/group. **g** Relative abundances of selected genera enriched by berberine. *n* = 12 (GH); *n* = 14 (GI, GIBBR). **h** Heatmap showing the relationships between different genera and morphological parameters of microglia. *n* = 6/group. *P < 0.05, **P < 0.01, ***P < 0.001, ****P < 0.0001. 1: Dendritic area; 2: dendritic length; 3: number of branch points; 4: number of segments; 5: number of terminal points.

Analysis of microbial community diversity showed significantly higher bacterial richness in the IBS-FMT group than in the HC-FMT group, and berberine decreased α-diversity in the IBS-FMT rats (Fig. 5a). Overall structural analysis of microbiota profiles revealed that there were significant differences between IBS-FMT rats and rats in the other two groups (Fig. 5b). Intriguingly, dynamic observations showed that the community structure of IBS-FMT rats began to shift towards that of HC-FMT rats 3 days after berberine treatment and remained stable until the end of the experiment (Fig. 5c). At the phylum level, Bacteroidetes and Firmicutes accounted for more than 90% of all phylogenetic types in each group. The relative abundance of Firmicutes was higher and that of Bacteroidetes was lower in IBS-FMT rats than in HC-FMT rats; berberine reversed these changes as well as the changes in the F/B ratio (Fig. 5d, e). These alterations also began from the third day of berberine treatment (Fig. 5f). At the genus level, alterations in several genera in IBS-FMT rats were rescued by berberine. Further analysis revealed that berberine enriched some SCFA-producing bacteria, including *Anaerostipes*, *Eubacterium*, *Lachnoclostridium*, and *Eisenbergiella*, and that the abundances of these bacteria were restored to those in HC-FMT rats (Fig. 5g), indicating that the therapeutic effects of berberine may be mediated by a subset of bacteria with specific functions. Furthermore, correlation analysis demonstrated that the abundances of the SCFA-producing bacteria *Eubacterium*, *Lachnoclostridium*, *Akkermansia*, and *Bifidobacterium* were positively correlated with microglial morphological parameters, suggesting that these bacteria may have protective effects on microglia (Fig. 5h).

### Berberine did not directly influence microglial activation

Since a very small amount of berberine can be absorbed into the circulation and enter the brain, we next investigated whether berberine could influence microglial activation by determining the berberine concentration in the brains of rats. Our results showed that the berberine concentration in the brain ranged from 0.04 to 0.2 μM. We then conducted an in vitro experiment to confirm the effects of berberine on microglial activation when used at the concentrations detected in rat brains. In this experiment, LPS induced the activation of microglia, and microglia transformed into an ameboid-like shape in the control group. Notably, when cells were treated with berberine at a concentration of 0.1 or 0.3 μM, this ameboid-like morphology was not reversed. However, when cells were treated with berberine at concentrations ranging from 1 to 30 μM, the original shape was restored in a concentration-dependent manner (Supplementary Fig. 3).

### Functions and metabolites of the gut microbiota were altered after berberine treatment

**Fig. 6:**
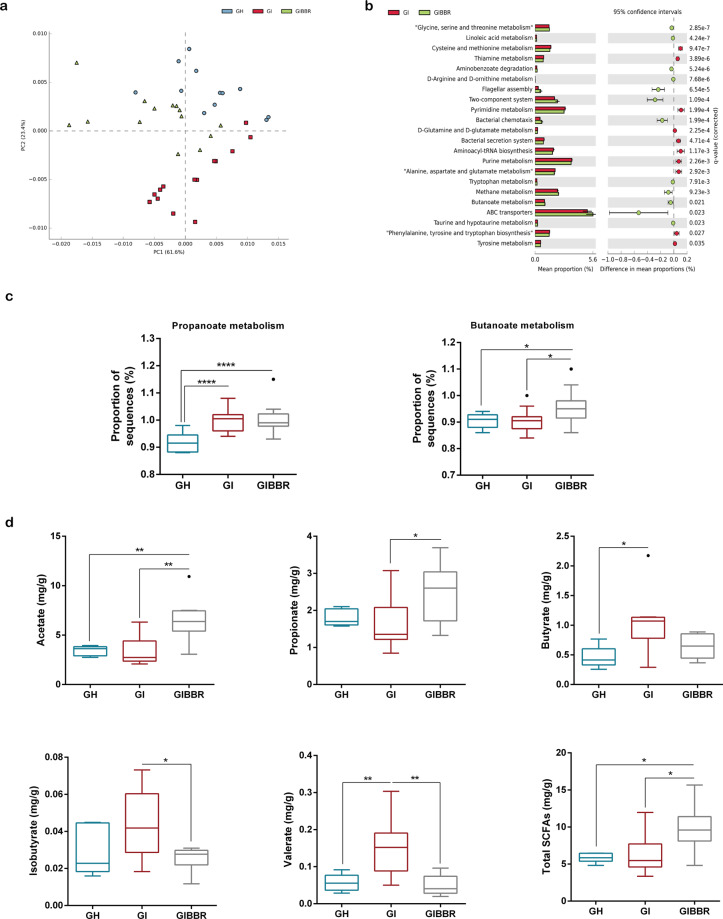
The functions and metabolites of the gut microbiota were altered after berberine treatment. **a** Principal component analysis of the enrichment of KEGG pathways. *n* = 12 (GH); *n* = 14 (GI, GIBBR). **b** Pathway enrichment analysis of significantly altered Kyoto Encyclopedia of Genes and Genomes orthologies after berberine treatment. *n* = 12 (GH); *n* = 14 (GI, GIBBR). **c** Enrichment of pathways involved in propanoate and butanoate. *n* = 12 (GH); *n* = 14 (GI, GIBBR). **d** Fecal concentrations of SCFAs. *n* = 6 (GH); *n* = 8 (GI, GIBBR). *P < 0.05, **P < 0.01, ****P < 0.0001.

To further identify functional changes in the gut microbiome after berberine treatment, we annotated genes identified by 16S rRNA sequencing according to Kyoto Encyclopedia of Genes and Genomes (KEGG) orthologies. Principal component analysis revealed obvious alterations in pathways in the berberine treatment group (Fig. 6a). Pathway enrichment analysis revealed that berberine treatment was linked mainly to the enrichment of genes involved in bacterial responses to the environment (flagellar assembly, two component system, bacterial chemotaxis, and ATP-binding cassette [ABC] transporters), amino acid metabolism (tryptophan, taurine, and hypotaurine), and carbohydrate metabolism (methane and butanoate; Fig. 6b).

Because berberine enriched both SCFA-producing bacteria (Figs. 2f, 5g) and genes related to SCFA (propanoate and butanoate) metabolism (Fig. 6c) in the gut microbiome, we sought to evaluate whether changes in SCFAs could be linked to the effects of berberine. We observed that acetate, propionate, and total SCFA concentrations were elevated, whereas valerate and isobutyrate concentrations were decreased after berberine treatment (Fig. 6d).

## Discussion

IBS is a disorder of the gut–brain interaction according to the ROME IV criteria [1]. The gut microbiota plays important roles in the pathogenesis of IBS, and berberine has been shown to be effective for IBS treatment. However, the mechanisms through which berberine influences the microbiota-gut-brain axis are poorly understood. In our study, we demonstrated that berberine altered the composition, function, and metabolites of the gut microbiota in both WAS (brain–gut pathway) and FMT (gut-brain pathway) models. Moreover, berberine enriched several SCFA-producing bacteria, thereby preventing microglial activation and improving visceral hypersensitivity.

Recent studies have shown that the ability of drugs to modulate the gut microbiota and alleviate disease symptoms could be transferred through fecal transplantation [44–47]. In our study, microbiota transfer to GF rats showed that colonization of the berberine-altered microbiota could prevent stress-induced visceral hypersensitivity, thus providing evidence for the key role of gut microbiota modulation in the therapeutic action of berberine.

In addition to the top-down pathway, gut dysbiosis has been shown to be an important mechanism in IBS pathogenesis, and recent studies have demonstrated the causal role of the gut microbiota in intestinal and behavioral manifestations [42, 43]. We previously found that differences in microbial structures were associated with the level of colon inflammation and the severity of symptoms in patients with IBS-D [31]. In the FMT experiment, the fecal microbiota from a donor with IBS-D caused visceral hypersensitivity accompanied by mast cell activation, further suggesting that the down-top pathway is a mechanism of IBS. Moreover, berberine could transform the structure of the microbiota in IBS-FMT GF rats to that of HC-FMT recipients, providing direct evidence for the regulatory effects of berberine on the gut microbiota. Based on these data, we concluded that alterations in the gut microbiota induced by berberine contributed to the therapeutic effects of the drug on visceral hypersensitivity and intestinal hypermotility.

In both stressed SPF rats and IBS-FMT gnotobiotic rats, berberine reduced gut bacterial α-diversity and the F/B ratio. Although the reason remains unclear, the F/B ratio is increased in patients with IBS [41, 48, 49] and is thought to be associated with alterations in gut epithelial permeability and low-grade inflammation [41], which are important mechanisms in IBS. The observation that berberine reversed the F/B ratio suggest that it is able to modulate the microbiota to a relatively healthy state.

By analyzing the microbiota abundance based on OTU data, we identified several genera that showed consistent changes after berberine administration in the two different models. In particular, the abundances of *Lachnoclostridium* and *Anaerostipes* were significantly lower in IBS-FMT rats than in HC-FMT rats and were enriched by berberine. A decrease in the relative abundance of *Lachnoclostridium* was observed in children with autism spectrum disorder, a psychiatric disorder characterized by comorbid gastrointestinal symptoms [50]. The abundance of *Lachnoclostridium* was also lower in dextran sulfate sodium-treated mice and was positively correlated with both vitamin B6 and tryptophan metabolism pathways [51], suggesting that it exerts protective effects against colitis. In particular, *Clostridium immunis*, a member of the genus *Lachnoclostridium*, has been found to protect against colitis [52]. Similarly, a study showed that the abundance of *Anaerostipes* was decreased in the feces of patients with IBS compared with the feces of healthy controls [53]. *Lachnoclostridium* and *Anaerostipes* are closely related to the production of butyrate, which has positive effects on gastrointestinal tract homeostasis by providing energy for the growth of intestinal epithelial cells, enhancing intestinal barrier function, and acting as an anti-inflammatory agent [54]. Hence, butyrate-producing bacteria are generally thought to promote the health of the host. Further studies are needed to determine whether *Lachnoclostridium* and *Anaerostipes* are beneficial for visceral hypersensitivity.

By analyzing fecal SCFAs, we found that in the FMT model, acetate and propionate concentrations were elevated in berberine-treated rats. However, the butyrate concentration did not differ between IBS-FMT rats and berberine-treated rats. The discrepancy between the enrichment of butyrate-producing genera and the lack of change in the butyrate concentration may be related to reductions in the abundances of other Firmicutes species. For example, in our study, the relative abundance of *Faecalibacterium*, a predominant butyrate-producing genus, was reduced from 21.64% ± 1.23% to 0.04% ± 0.03% after berberine treatment. The extent of this change was much larger than the changes observed for *Lachnoclostridium* and *Anaerostipes* and may have caused the overall butyrate concentration to remain unchanged. The two predominant phyla, Bacteroidetes and Firmicutes, exist in a careful balance whereby Firmicutes can utilize acetate from Bacteroidetes to produce butyrate [55]. Therefore, the elevation of acetate concentration may have been caused by decreases in the levels of several bacteria that use acetate as a substrate and enrichment of acetate-producing bacteria, as reflected by the increased gene expression of ABC transporters in the berberine group, which is required for acetate production [56]. Therefore, berberine-dependent alterations in the microbial balance and the complex interactions within the microbiota may cooperate to modulate the production of SCFAs.

Many studies have shown that the gut microbiota has an important impact on brain development and function, including microglia, the key player in brain–immune interactions. As brain resident macrophages, microglial cells are activated and transform from a highly branched and ramified morphology to an ameboid-like shape when exposed to adverse stimuli, such as stress and pathogens. Microglia have been reported to be crucial in the microbiota–gut–brain axis. Positron emission tomography scanning showed increased microglial activation in patients with major depressive disorder [57] and autism spectrum disorder [58]. Notably, the incidence of psychological comorbidities, particularly anxiety and depression, is high in patients with IBS [59], and antidepression therapy can effectively relieve the clinical symptoms of refractory IBS [60]. Although there is no direct evidence showing that microglia in the CNS of patients with IBS are activated, several animal experiments have found that microglia in the spinal cord and hippocampus play roles in visceral hypersensitivity caused by stress or chemical colitis [17, 61, 62]. For example, intrathecal injection of fractalkine (an agonist of microglia) enhances visceral nociception, whereas percutaneous injection of minocycline (an inhibitor of microglia) inhibits visceral hypersensitivity in chronically sensitized rats [63]. To the best of our knowledge, our study shows, for the first time, that the fecal microbiota of a patient with IBS-D induced microglial priming, which was rescued by modulation of the microbiota by berberine. In addition, berberine reversed morphological changes in microglia and alleviated depressive behaviors in IBS-FMT rats. We also found that berberine inhibited stress-induced microglial activation. Erny et al. [64] first showed that the gut microbiota and its metabolites control the maturation and functions of microglia, and therapeutic methods targeting the gut microbiota, including probiotics, prebiotics, SCFAs, and FMT, have recently been shown to have protective effects on microglial activation [22–24]. The beneficial role of berberine may be related to the enrichment of SCFA-producing and probiotic bacteria, as shown by the positive correlations of microglial parameters with *Eubacterium*, *Lachnoclostridium*, *Eisenbergiella*, *Akkermansia*, and *Bifidobacterium*. Another possible mechanistic explanation is the elevation of acetate concentrations. Acetate is able to cross the blood-brain barrier and is taken up by the brain. Frost et al. revealed that hypothalamic acetate regionally increases the levels of glutamate-glutamine and GABA neuroglial cycles [65], and administration of the probiotic *Clostridium butyricum* or the prebiotic Hylon VII to rats fed a high-fat diet reduces microglial activation accompanied by increases in cecal or plasma acetate concentrations [66].

Moreover, to exclude the direct influence of berberine on microglia, we determined the concentration of berberine in the brain and conducted an in vitro experiment. The results suggested that treatment with berberine at the physiological concentration reached in the rat brain did not influence the activation of microglial cells, providing indirect evidence that berberine inhibits the activation of microglial cells through the microbiota-gut-brain axis, not via direct effects.

**Fig. 7:**
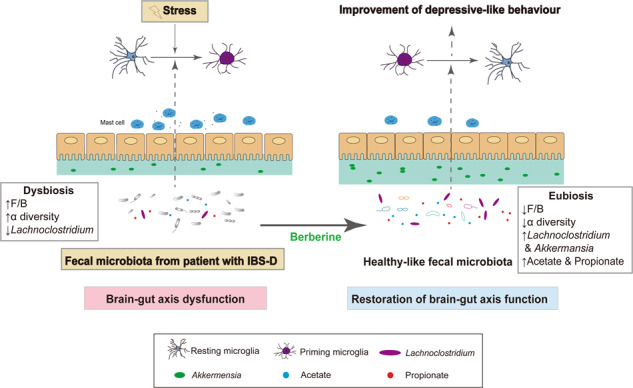
Proposed model of the beneficial effects of berberine on gut dysbiosis and stress-induced brain–gut dysregulation.

In summary, our work revealed that microglia were activated both by stress and the fecal microbiota of a patient with IBS and that these effects were prevented by berberine treatment. Treatment targeting the gut microbiota with berberine modulated the composition, function and metabolites of the gut microbiota and alleviated visceral hypersensitivity, intestinal hypermotility, and depressive behaviors in models of both brain–gut and gut–brain mechanisms, thereby blocking microglial activation (Fig. 7). Our findings show that microglia are the key targets in the pathogenesis of IBS and that the regulation of the microbiota–gut–brain axis may represent a potential therapeutic method for IBS, particularly in patients with concurrent IBS and mental disorders. Further studies are required to clarify the mechanisms through which berberine-enriched bacteria affect microglial activation in visceral hypersensitivity. Moreover, clinical trials are warranted to verify the effects of berberine on the microbiota–gut–brain axis in patients with IBS.

## Supplementary information

Supplementary Information

## References

[1] Drossman DA, Hasler WL (2016). Rome IV-functional GI disorders: disorders of gut-brain interaction. Gastroenterology..

[2] Ford AC, Lacy BE, Talley NJ (2017). Irritable bowel syndrome. N Engl J Med.

[3] Koloski NA, Jones M, Talley NJ (2016). Evidence that independent gut-to-brain and brain-to-gut pathways operate in the irritable bowel syndrome and functional dyspepsia: a 1-year population-based prospective study. Aliment Pharmacol Ther..

[4] Moloney RD, O’Mahony SM, Dinan TG, Cryan JF (2015). Stress-induced visceral pain: toward animal models of irritable-bowel syndrome and associated comorbidities. Front Psychiatry.

[5] Wilcz-Villega EM, McClean S, O’Sullivan MA (2013). Mast cell tryptase reduces junctional adhesion molecule-A (JAM-A) expression in intestinal epithelial cells: implications for the mechanisms of barrier dysfunction in irritable bowel syndrome. Am J Gastroenterol.

[6] Piche T (2014). Tight junctions and IBS–the link between epithelial permeability, low-grade inflammation, and symptom generation?. Neurogastroenterol Motil.

[7] Simren M, Tornblom H, Palsson OS, van Tilburg MAL, Van Oudenhove L, Tack J (2018). Visceral hypersensitivity is associated with GI symptom severity in functional GI disorders: consistent findings from five different patient cohorts. Gut..

[8] Tornblom H, Van Oudenhove L, Sadik R, Abrahamsson H, Tack J, Simren M (2012). Colonic transit time and IBS symptoms: what’s the link?. Am J Gastroenterol.

[9] Jeffery IB, O’Toole PW, Ohman L, Claesson MJ, Deane J, Quigley EMM (2012). An irritable bowel syndrome subtype defined by species-specific alterations in faecal microbiota. Gut..

[10] Tap J, Derrien M, Tornblom H, Brazeilles R, Cools-Portier S, Dore J (2017). Identification of an intestinal microbiota signature associated with severity of irritable bowel syndrome. Gastroenterology..

[11] Collins SM (2014). A role for the gut microbiota in IBS. Nat Rev Gastroenterol Hepatol.

[12] Eisenstein M (2016). Microbiome: bacterial broadband. Nature..

[13] Sampson TR, Mazmanian SK (2015). Control of brain development, function, and behavior by the microbiome. Cell Host Microbe.

[14] Bennet SM, Polster A, Tornblom H, Isaksson S, Capronnier S, Tessier A (2016). Global cytokine profiles and association with clinical characteristics in patients with irritable bowel syndrome. Am J Gastroenterol.

[15] Chang L, Adeyemo M, Karagiannides I, Videlock EJ, Bowe C, Shih W (2012). Serum and colonic mucosal immune markers in irritable bowel syndrome. Am J Gastroenterol.

[16] Wolf SA, Boddeke HW, Kettenmann H (2017). Microglia in physiology and disease. Annu Rev Physiol.

[17] Bradesi S, Svensson CI, Steinauer J, Pothoulakis C, Yaksh TL, Mayer EA (2009). Role of spinal microglia in visceral hyperalgesia and NK1R up-regulation in a rat model of chronic stress. Gastroenterology..

[18] Liu PY, Lu CL, Wang CC, Lee IH, Hsieh JC, Chen CC (2012). Spinal microglia initiate and maintain hyperalgesia in a rat model of chronic pancreatitis. Gastroenterology..

[19] Zhang G, Yu L, Chen ZY, Zhu JS, Hua R, Qin X (2016). Activation of corticotropin-releasing factor neurons and microglia in paraventricular nucleus precipitates visceral hypersensitivity induced by colorectal distension in rats. Brain Behav Immun.

[20] Sampson TR, Debelius JW, Thron T, Janssen S, Shastri GG, Ilhan ZE (2016). Gut microbiota regulate motor deficits and neuroinflammation in a model of Parkinson’s disease. Cell..

[21] Liu R, Kang JD, Sartor RB, Sikaroodi M, Fagan A, Gavis EA (2019). Neuroinflammation in murine cirrhosis is dependent on the gut microbiome and is attenuated by fecal transplant. Hepatology..

[22] Chunchai T, Thunapong W, Yasom S, Wanchai K, Eaimworawuthikul S, Metzler G (2018). Decreased microglial activation through gut-brain axis by prebiotics, probiotics, or synbiotics effectively restored cognitive function in obese-insulin resistant rats. J Neuroinflammation.

[23] Matt SM, Allen JM, Lawson MA, Mailing LJ, Woods JA, Johnson RW (2018). Butyrate and dietary soluble fiber improve neuroinflammation associated with aging in mice. Front Immunol.

[24] Sun MF, Zhu YL, Zhou ZL, Jia XB, Xu YD, Yang Q (2018). Neuroprotective effects of fecal microbiota transplantation on MPTP-induced Parkinson’s disease mice: Gut microbiota, glial reaction and TLR4/TNF-alpha signaling pathway. Brain Behav Immun.

[25] Wang Y, Tong Q, Shou JW, Zhao ZX, Li XY, Zhang XF (2017). Gut microbiota-mediated personalized treatment of hyperlipidemia using berberine. Theranostics..

[26] Kulkarni SK, Dhir A (2010). Berberine: a plant alkaloid with therapeutic potential for central nervous system disorders. Phytother Res.

[27] Chen YX, Gao QY, Zou TH, Wang BM, Liu SD, Sheng JQ (2020). Berberine versus placebo for the prevention of recurrence of colorectal adenoma: a multicentre, double-blinded, randomised controlled study. Lancet Gastroenterol Hepatol..

[28] Zhang X, Zhao Y, Xu J, Xue Z, Zhang M, Pang X (2015). Modulation of gut microbiota by berberine and metformin during the treatment of high-fat diet-induced obesity in rats. Sci Rep.

[29] Zhang X, Zhao Y, Zhang M, Pang X, Xu J, Kang C (2012). Structural changes of gut microbiota during berberine-mediated prevention of obesity and insulin resistance in high-fat diet-fed rats. PLoS One.

[30] Xu D, Gao J, Gillilland M, Wu X, Song I, Kao JY (2014). Rifaximin alters intestinal bacteria and prevents stress-induced gut inflammation and visceral hyperalgesia in rats. Gastroenterology..

[31] Liu Y, Zhang L, Wang X, Wang Z, Zhang J, Jiang R (2016). Similar fecal microbiota signatures in patients with diarrhea-predominant irritable bowel syndrome and patients with depression. Clin Gastroenterol Hepatol.

[32] Hong S, Fan J, Kemmerer ES, Evans S, Li Y, Wiley JW (2009). Reciprocal changes in vanilloid (TRPV1) and endocannabinoid (CB1) receptors contribute to visceral hyperalgesia in the water avoidance stressed rat. Gut..

[33] Al-Chaer ED, Kawasaki M, Pasricha PJ (2000). A new model of chronic visceral hypersensitivity in adult rats induced by colon irritation during postnatal development. Gastroenterology..

[34] Snyder JS, Soumier A, Brewer M, Pickel J, Cameron HA (2011). Adult hippocampal neurogenesis buffers stress responses and depressive behaviour. Nature..

[35] Xie W, Cai L, Yu Y, Gao L, Xiao L, He Q (2014). Activation of brain indoleamine 2,3-dioxygenase contributes to epilepsy-associated depressive-like behavior in rats with chronic temporal lobe epilepsy. J Neuroinflammation.

[36] Seminowicz DA, Labus JS, Bueller JA, Tillisch K, Naliboff BD, Bushnell MC (2010). Regional gray matter density changes in brains of patients with irritable bowel syndrome. Gastroenterology..

[37] Wouters MM, Van Wanrooy S, Casteels C, Nemethova A, de Vries A, Van Oudenhove L (2012). Altered brain activation to colorectal distention in visceral hypersensitive maternal-separated rats. Neurogastroenterol Motil.

[38] Zhao J, Zhang QL, Shen JH, Wang K, Liu J (2019). Magnesium lithospermate B improves the gut microbiome and bile acid metabolic profiles in a mouse model of diabetic nephropathy. Acta Pharmacol Sin..

[39] Amato KR, Yeoman CJ, Kent A, Righini N, Carbonero F, Estrada A (2013). Habitat degradation impacts black howler monkey (*Alouatta pigra*) gastrointestinal microbiomes. ISME J..

[40] Lu DY, Tang CH, Chen YH, Wei IH (2010). Berberine suppresses neuroinflammatory responses through AMP-activated protein kinase activation in BV-2 microglia. J Cell Biochem.

[41] Labus JS, Hollister EB, Jacobs J, Kirbach K, Oezguen N, Gupta A (2017). Differences in gut microbial composition correlate with regional brain volumes in irritable bowel syndrome. Microbiome..

[42] Crouzet L, Gaultier E, Del’Homme C, Cartier C, Delmas E, Dapoigny M (2013). The hypersensitivity to colonic distension of IBS patients can be transferred to rats through their fecal microbiota. Neurogastroenterol Motil.

[43] De Palma G, Lynch MD, Lu J, Dang VT, Deng Y, Jury J (2017). Transplantation of fecal microbiota from patients with irritable bowel syndrome alters gut function and behavior in recipient mice. Sci Transl Med..

[44] Anhe FF, Nachbar RT, Varin TV, Trottier J, Dudonne S, Le Barz M (2018). Treatment with camu camu (*Myrciaria dubia*) prevents obesity by altering the gut microbiota and increasing energy expenditure in diet-induced obese mice. Gut..

[45] Wu H, Esteve E, Tremaroli V, Khan MT, Caesar R, Manneras-Holm L (2017). Metformin alters the gut microbiome of individuals with treatment-naive type 2 diabetes, contributing to the therapeutic effects of the drug. Nat Med..

[46] Liao X, Song L, Zeng B, Liu B, Qiu Y, Qu H (2019). Alteration of gut microbiota induced by DPP-4i treatment improves glucose homeostasis. EBioMedicine..

[47] Chang CJ, Lin CS, Lu CC, Martel J, Ko YF, Ojcius DM (2015). Ganoderma lucidum reduces obesity in mice by modulating the composition of the gut microbiota. Nat Commun.

[48] Rajilic-Stojanovic M, Biagi E, Heilig HG, Kajander K, Kekkonen RA, Tims S (2011). Global and deep molecular analysis of microbiota signatures in fecal samples from patients with irritable bowel syndrome. Gastroenterology..

[49] Chung CS, Chang PF, Liao CH, Lee TH, Chen Y, Lee YC (2016). Differences of microbiota in small bowel and faeces between irritable bowel syndrome patients and healthy subjects. Scand J Gastroenterol.

[50] Ma B, Liang J, Dai M, Wang J, Luo J, Zhang Z (2019). Altered gut microbiota in Chinese children with autism spectrum disorders. Front Cell Infect Microbiol.

[51] Haange SB, Jehmlich N, Hoffmann M, Weber K, Lehmann J, von Bergen M (2019). Disease development is accompanied by changes in bacterial protein abundance and functions in a refined model of dextran sulfate sodium (DSS)-induced colitis. J Proteome Res.

[52] Surana NK, Kasper DL (2017). Moving beyond microbiome-wide associations to causal microbe identification. Nature..

[53] Lo Presti A, Zorzi F, Del Chierico F, Altomare A, Cocca S, Avola A (2019). Fecal and mucosal microbiota profiling in irritable bowel syndrome and inflammatory bowel disease. Front Microbiol.

[54] Tan J, McKenzie C, Potamitis M, Thorburn AN, Mackay CR, Macia L (2014). The role of short-chain fatty acids in health and disease. Adv Immunol.

[55] Mahowald MA, Rey FE, Seedorf H, Turnbaugh PJ, Fulton RS, Wollam A (2009). Characterizing a model human gut microbiota composed of members of its two dominant bacterial phyla. Proc Natl Acad Sci USA.

[56] Fukuda S, Toh H, Hase K, Oshima K, Nakanishi Y, Yoshimura K (2011). Bifidobacteria can protect from enteropathogenic infection through production of acetate. Nature..

[57] Setiawan E, Wilson AA, Mizrahi R, Rusjan PM, Miler L, Rajkowska G (2015). Role of translocator protein density, a marker of neuroinflammation, in the brain during major depressive episodes. JAMA Psychiatry..

[58] Suzuki K, Sugihara G, Ouchi Y, Nakamura K, Futatsubashi M, Takebayashi K (2013). Microglial activation in young adults with autism spectrum disorder. JAMA Psychiatry.

[59] Fond G, Loundou A, Hamdani N, Boukouaci W, Dargel A, Oliveira J (2014). Anxiety and depression comorbidities in irritable bowel syndrome (IBS): a systematic review and meta-analysis. Eur Arch Psychiatry Clin Neurosci.

[60] Ford AC, Quigley EM, Lacy BE, Lembo AJ, Saito YA, Schiller LR (2014). Effect of antidepressants and psychological therapies, including hypnotherapy, in irritable bowel syndrome: systematic review and meta-analysis. Am J Gastroenterol.

[61] Basso L, Lapointe TK, Iftinca M, Marsters C, Hollenberg MD, Kurrasch DM (2017). Granulocyte-colony-stimulating factor (G-CSF) signaling in spinal microglia drives visceral sensitization following colitis. Proc Natl Acad Sci USA.

[62] Zhang G, Zhao BX, Hua R, Kang J, Shao BM, Carbonaro TM (2016). Hippocampal microglial activation and glucocorticoid receptor down-regulation precipitate visceral hypersensitivity induced by colorectal distension in rats. Neuropharmacology..

[63] Saab CY, Wang J, Gu C, Garner KN, Al-Chaer ED (2007). Microglia a newly discovered role in visceral hypersensitivity. Neuron Glia Biol..

[64] Erny D, Hrabe de Angelis AL, Jaitin D, Wieghofer P, Staszewski O, David E (2015). Host microbiota constantly control maturation and function of microglia in the CNS. Nat Neurosci.

[65] Frost G, Sleeth ML, Sahuri-Arisoylu M, Lizarbe B, Cerdan S, Brody L (2014). The short-chain fatty acid acetate reduces appetite via a central homeostatic mechanism. Nat Commun.

[66] Ganesh BP, Nelson JW, Eskew JR, Ganesan A, Ajami NJ, Petrosino JF (2018). Prebiotics, probiotics, and acetate supplementation prevent hypertension in a model of obstructive sleep apnea. Hypertension..

