# Supplementary material for: Berberine alleviates visceral hypersensitivity in rats by altering gut microbiome and suppressing spinal microglial activation
Source: Acta Pharmacol Sin. 2021 Feb 8;42(11):1821–33. doi: 10.1038/s41401-020-00601-4 (PMC8563748; doi:10.1038/s41401-020-00601-4)
Supplement: Supplementary file 1 — Supplementary Information [file 41401_2020_601_MOESM1_ESM.docx]

**
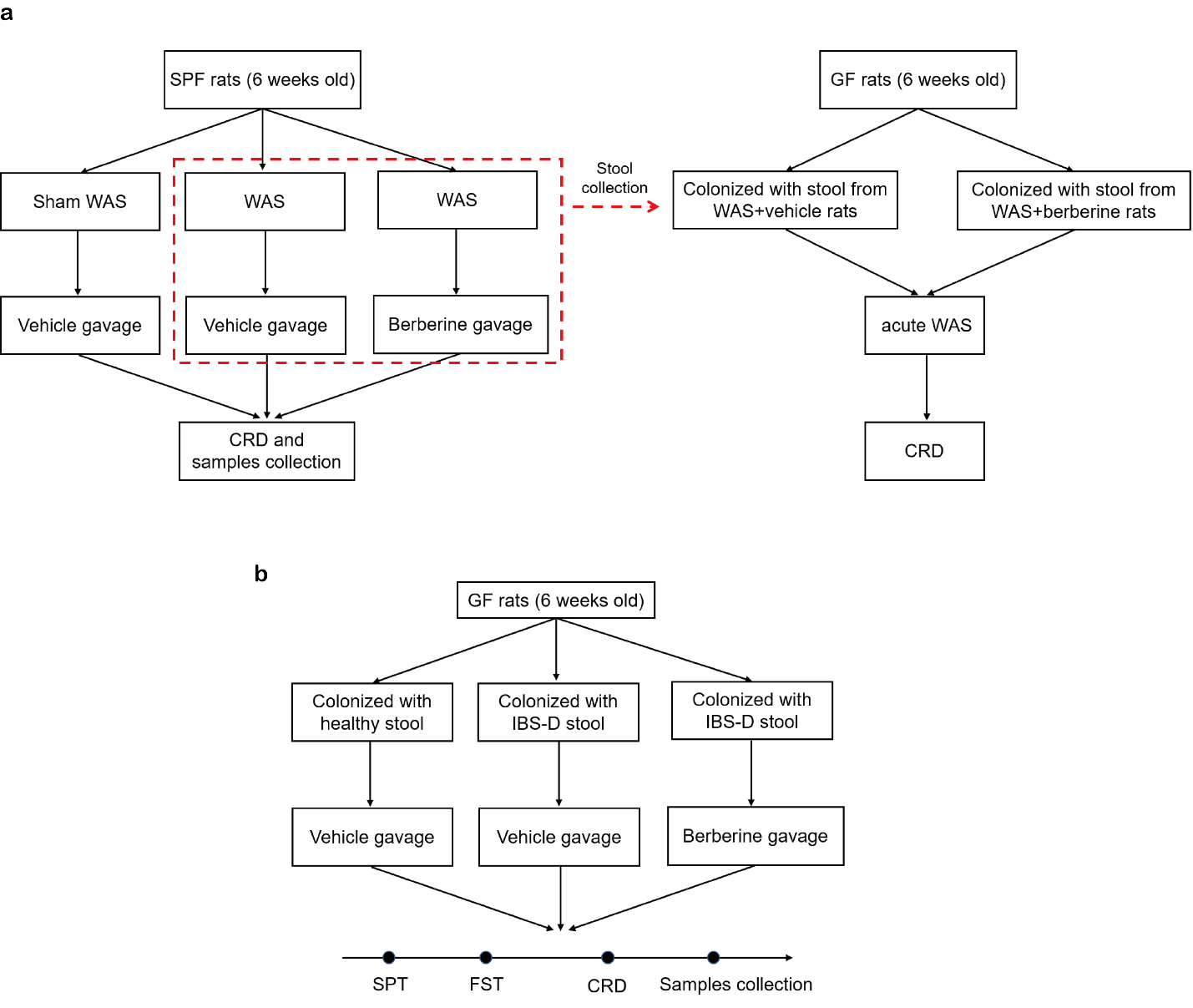
**

**Supplementary Figure 1.** Schematic of experiments in the WAS model (a) and FMT model (b).

**
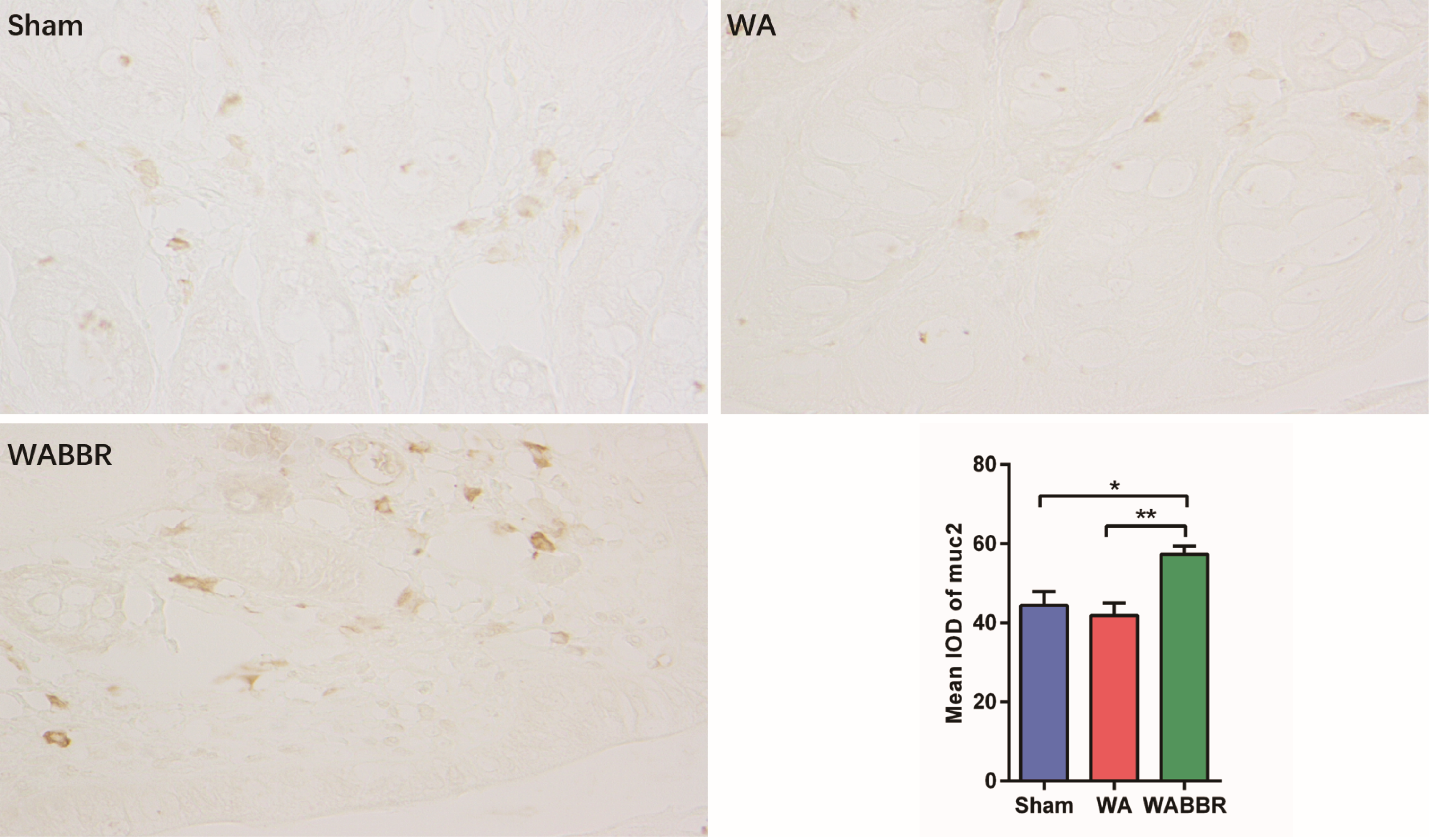
**

**Supplementary Figure 2.** Berberine increased the expression of Muc2 in the colonic mucus layer. **P* < 0.05, ** *P* < 0.01.


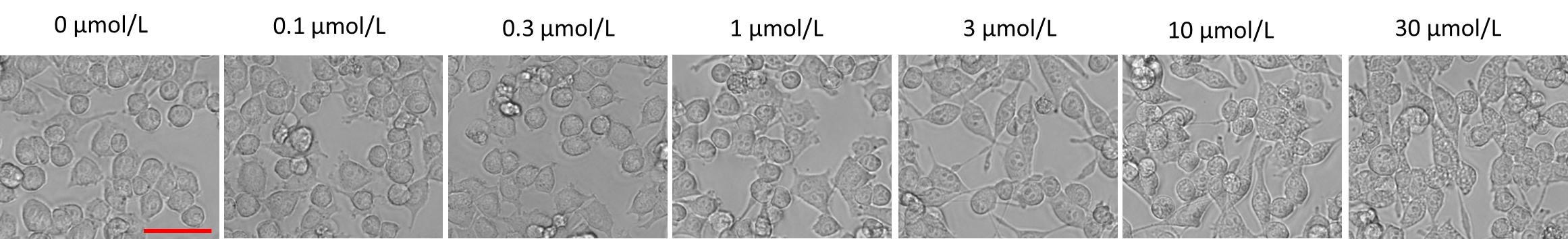


**Supplementary Figure 3.** Effect of berberine at different concentration on LPS-induced activation of microglia cells. Representative pictures are from three independent and repeated experiment. Scale bar = 50 μm.

**Supplemental Table 1.** Demographics of Human Donors

| Donor | Gender | Dietary preferences | IBS-SSS | SAS | SDS | Microbiota-related type |
| --- | --- | --- | --- | --- | --- | --- |
| HC | Female | Vegetarian | 0 | 27 | 27 | Type III |
| IBS-D | Female | Vegetarian | 205 | 47 | 48 | Type I |

HC, healthy control; IBS-SSS, IBS symptom severity score; SAS, self-rating anxiety scale; SDS, self-rating depression scale. Type I, Bacteroides-predominant; Type III, Bacteroides/Prevotella balanced.

**Supplemental Table 2.** Number of Iba-1 positive cells in the selected CNS regions in the WAS experiment (numbers per 0.01 mm^2^).

| Regions | Sham | WA | WABBR |
| --- | --- | --- | --- |
| Hippocampus | 1.64±0.28 | 2.28±0.29 | 1.93±0.04 |
| Amygdala | 2.40±0.07 ^a^ | 3.84±0.19 | 2.68±0.11 ^a^ |
| Prefrontal cortex | 1.53±0.10 | 2.37±0.24 | 1.96±0.12 |
| Spinal cord | 1.18±0.08 ^a^ | 1.83±0.10 | 1.38±0.08 |

^a^ *P* < 0.05 compared with WA group.

**Supplemental Table 3.** Morphological parameters of microglia in the selected CNS regions in the WAS experiment.

| Regions | Parameters | Sham | WA | WABBR |
| --- | --- | --- | --- | --- |
| Hippocampus | Area（μm^2^） | 1319.6±149.2 | 1083.0±69.4 | 1291.9±59.9 |
|  | Length（μm） | 472.0±49.2 ^a^ | 336.6±24.0 | 380.2±19.5 |
|  | Branch points | 33.1± 5.1 | 24.3± 2.3 | 24.6± 2.0 |
|  | Segments | 72.3± 10.9 | 53.0± 4.9 | 53.1± 4.1 |
|  | Terminal points | 39.2±5.8 | 28.8±2.5 | 28.5± 2.1 |
| Amygdala | Area（μm^2^） | 1149.9± 94.7 | 961.9± 56.0 | 1259.3± 96.6 ^a^ |
|  | Length（μm） | 400.3± 39.8 | 303.0± 16.6 | 401.1± 33.6 ^a^ |
|  | Branch points | 27.9± 4.0 | 22.9± 1.8 | 27.2± 3.1 |
|  | Segments | 61.8± 8.6 | 50.8± 3.6 | 59.9± 6.5 |
|  | Terminal points | 33.9± 4.7 | 27.9± 1.9 | 32.7± 3.5 |
| Prefrontal cortex | Area（μm^2^） | 1110.3± 63.7 | 953.2± 52.0 | 1124.3± 56.9 |
|  | Length（μm） | 394.9± 25.6 ^a^ | 290.2± 16.1 | 356.6± 18.7 ^a^ |
|  | Branch points | 29.3± 2.6 ^a^ | 18.3± 1.5 | 23.3± 2.0 |
|  | Segments | 63.6± 5.4 ^a^ | 41.5± 3.2 | 52.2± 4.1 |
|  | Terminal points | 34.3± 2.8 ^a^ | 23.2± 1.7 | 28.9± 2.2 |
| Spinal cord | Area（μm^2^） | 1022.9± 74.6 ^a^ | 733.8± 49.4 | 1092.9± 70.1 ^a^ |
|  | Length（μm） | 330.1± 26.7 ^a^ | 194.4± 11.4 | 307.6± 22.2 ^a^ |
|  | Branch points | 22.0± 3.3 ^a^ | 11.5± 1.1 | 22.0± 2.3 ^a^ |
|  | Segments | 47.9± 6.9 ^a^ | 26.0± 2.4 | 47.5± 4.7 ^a^ |
|  | Terminal points | 26.9± 3.6 ^a^ | 14.5± 1.3 | 25.6± 2.5 ^a^ |

^a^ *P* < 0.05 compared with WA group.

**Supplemental Table 4.** Number of Iba-1 positive cells in the selected CNS regions in the FMT experiment (numbers per 0.01 mm^2^).

| Regions | GH | GI | GIBBR |
| --- | --- | --- | --- |
| Hippocampus | 1.70±0.23 | 2.16±0.16 | 1.67±0.11 |
| Amygdala | 2.17±0.18 | 2.76±0.19 | 2.20±0.18 |
| Prefrontal cortex | 2.20±0.29 | 2.17±0.14 | 1.97±0.10 |
| Spinal cord | 1.65±0.14 | 1.93±0.23 | 1.72±0.23 |

**Supplemental Table 5.** Morphological parameters of microglia in the selected CNS regions in the FMT experiment.

| Regions | Parameters | GH | GI | GIBBR | |
| --- | --- | --- | --- | --- | --- |
| Hippocampus | Area（μm^2^） | 1945.9±290.4 | 1134.0±58.0 | 1991.6±283.4 | |
|  | Length（μm） | 589.3±84.2 ^a^ | 329.9±20.1 | 560.3±56.3 ^a^ |  |
|  | Branch points | 44.5± 7.8 ^a^ | 22.5± 1.1 | 41.1± 4.4 | |
|  | Segments | 94.3± 16.0 ^a^ | 48.8± 2.2 | 87.3± 9.1 | |
|  | Terminal points | 49.9±8.2 ^a^ | 26.3±1.1 | 46.1± 4.7 ^a^ | |
| Amygdala | Area（μm^2^） | 1933.2±186.7 ^a^ | 1052.2±67.8 | 1959.3±318.1 ^a^ | |
|  | Length（μm） | 664.6± 63.9 ^a^ | 336.6± 24.6 | 534.2± 61.6 ^a^ | |
|  | Branch points | 50.6± 5.3 ^a^ | 25.8± 2.9 | 40.7± 4.9 | |
|  | Segments | 106.9± 10.9 ^a^ | 55.7±5.9 | 86.5± 10.3 | |
|  | Terminal points | 56.4± 5.7 ^a^ | 30.5± 3.3 | 45.8± 5.4 | |
| Prefrontal cortex | Area（μm^2^） | 1952.2±244.1^a^ | 959.5± 47.0 | 1648.7±200.4 ^a^ | |
|  | Length（μm） | 581.4± 63.2 ^a^ | 283.0± 9.9 | 472.3± 29.1 ^a^ | |
|  | Branch points | 44.8± 5.5 ^a^ | 20.0± 0.6 | 35.1± 2.5 ^a^ | |
|  | Segments | 95.0± 11.6 ^a^ | 43.8± 1.2 | 75.0± 5.1 ^a^ | |
|  | Terminal points | 50.3± 6.1 ^a^ | 23.8± 0.6 | 39.9± 2.7 ^a^ | |
| Spinal cord | Area（μm^2^） | 1144.6±119.3 ^a^ | 665.8± 58.1 | 1164.9±121.1 ^a^ | |
|  | Length（μm） | 288.1±33.9 ^a^ | 165.3± 11.5 | 282.9± 15.4 ^a^ | |
|  | Branch points | 20.0± 3.1 ^a^ | 10.6± 0.6 | 18.6± 0.8 ^a^ | |
|  | Segments | 43.2± 6.4 ^a^ | 23.9± 1.4 | 40.7± 1.7 ^a^ | |
|  | Terminal points | 23.1± 3.3 ^a^ | 13.3± 0.8 | 22.1± 0.9 ^a^ | |

^a^ *P* <.05 compared with GI group.
